# Vitamins A and E during Pregnancy and Allergy Symptoms in an Early Childhood—Lack of Association with Tobacco Smoke Exposure

**DOI:** 10.3390/ijerph15061245

**Published:** 2018-06-12

**Authors:** Jolanta Gromadzinska, Kinga Polanska, Lucyna Kozlowska, Karolina Mikolajewska, Iwona Stelmach, Joanna Jerzynska, Wlodzimierz Stelmach, Mariusz Grzesiak, Wojciech Hanke, Wojciech Wasowicz

**Affiliations:** 1 Department of Biological and Environmental Monitoring, Nofer Institute of Occupational Medicine, 91-348 Lodz, Poland; karolina.mikolajewska@imp.lodz.pl (K.M.); wojciech.wasowicz@imp.lodz.pl (W.W.); 2 Department of Environmental Epidemiology, Nofer Institute of Occupational Medicine, 91-348 Lodz, Poland; kinga.polanska@imp.lodz.pl (K.P.); wojciech.hanke@imp.lodz.pl (W.H.); 3 Department of Dietetics, Faculty of Human Nutrition, 02-776 Warsaw, Poland; lucyna_kozlowska@sggw.pl; 4 Department of Pediatrics and Allergy, Medical University of Lodz, 90-329 Lodz, Poland; iwona.stelmach@umed.lodz.pl (I.S.); joanna.jerzynska@umed.lodz.pl (J.J.); 5 Department of Social and Preventive Medicine, Medical University of Lodz, 90-647 Lodz, Poland; wlodzimierz.stelmach@umed.lodz.pl; 6 Obstetrics, Perinatology and Gynecology Department, Polish Mother’s Memorial Hospital Research Institute, 93-338 Lodz, Poland; mariusz.grzesiak@gmail.com

**Keywords:** vitamins A, E, β-carotene, pregnancy, smoking, dermal, respiratory and food allergy, children

## Abstract

Epidemiological studies have suggested an association between maternal antioxidant levels during pregnancy and development of allergic diseases in their offspring. The aim of the study was to determine plasma vitamins A and E concentration in the 1st trimester of pregnancy, at delivery and in cord blood and to search for a relationship with allergy in up to 2-year-old children who were prenatally exposed or not exposed to tobacco smoke. The study participants included 252 mother-child pairs from Polish Mother and Child Cohort. Vitamin concentrations were measured using the HPLC-UV method, smoking status—as saliva cotinine level using the HPLC-MS/MS technique. Children’s health status was assessed using a questionnaire and pediatricians/allergists examination. Cord plasma vitamin concentrations were significantly lower than their levels in maternal plasma in the 1st trimester and at delivery (*p* < 0.001). Significantly higher concentrations of vitamin E have been shown to occur during the 1st trimester of pregnancy in plasma of the women who have actively/passively smoked cigarettes compared to the non-smokers (*p* < 0.02). Multivariate analysis with inclusion of a variety of confounding factors have not indicated any statistically significant associations between β-carotene, vitamins A and E and the risk of food allergy, atopic dermatitis and wheezing in their children up to 2 years of age. The interaction between smoking during pregnancy and vitamins levels on the risk of allergy was not statistically significant (*p* > 0.4). The relationship between plasma concentration of vitamins A and E, and the risk of allergy in their young children has not been demonstrated.

## 1. Introduction

Allergic diseases constitute an increasing health problem; from year to year their frequency is increasing. Numerous studies on that subject have been undertaken and a large number of risk factors have been identified [1]. It is believed that the development of allergic diseases is a result of interaction between genetic predispositions, air pollution, lifestyle and environmental chemical exposure [2,3]. Exposure of pregnant women to air pollutants (particulate matter: PM_2.5_, PM_10_, nitrogen oxides, ozone, diesel exhaust), environmental irritants (titanium oxide) or skin sensitization (toluene diisocyanate) can affect the risk of allergy development in their children [4]. It is believed that prenatal and postnatal exposure to tobacco smoke is a factor that contributes to the development of not only respiratory but also dermal allergies in infants and young children [5].

Pregnancy is potentially associated with an increasing risk of oxidative stress [6]. Increased reactive oxygen species (ROS) production is normal in the second and third trimesters of pregnancy and is accompanied by elevated markers of oxidative stress [7,8]. Overproduction of ROS has been suggested to be involved in pregnancy-related disorders, such as: decreased lung function, hypertension, preeclampsia, spontaneous abortion, low for gestational age, preterm delivery or fetal death [9,10]. There are a few studies suggesting that cigarette smoking during pregnancy is an additional source of oxidative stress for mothers and their children [11]. There is plenty of evidence that allergic and inflammatory skin diseases are mediated by oxidative stress, and that development of respiratory allergy is associated with generation of oxidative stress [12,13,14].

Cook-Mills, when analyzing a number of both epidemiological and experimental animal studies, indicates that in humans and in animal models development of allergic diseases may start in the prenatal or early postnatal life. A link has been shown to occur between the increased risk of eczema, wheezing and lower respiratory tract infections in the first months after birth and the presence of acute phase proteins in the venous blood of an allergic mother and in umbilical cord blood [4].

The role of some vitamins intake by pregnant women with a diet or in a form of dietary supplements in the development of allergies in their children has not been completely explained. Vitamins A, C and E have a proven antioxidant activity. Their role in the development of allergy has been widely studied; however, it has not been completely explained [15,16]. Literature reviews show that pregnant women who smoke have lower concentrations of low-molecular-weight antioxidants than non-smokers [17]. Allergic diseases associated with the respiratory tract (asthma, rhinitis) develop in younger children, while the risk of eczema development usually occurs in older age, up to 16 years of age [18]. Since contribution of low molecular antioxidants to the development of young children allergy is considered, it seemed advisable to evaluate the potential link between development of allergies and the level of antioxidants twice—separately for children in the first year of life and for the two-year-old.

The aim of the study was to determine concentration of β-carotene (a precursor of vitamin A), vitamins A and E in the venous blood collected in the 1st trimester of pregnancy, at delivery and in cord and to search for a relationship with dermal, respiratory and food allergy in up to 2-year-old children who were prenatally exposed and were not exposed to tobacco smoke.

## 2. Material and Methods

### 2.1. Study Design and Population

The study subjects constituted a sample of the population from Polish Mother and Child Cohort (REPRO_PL). A complete description of the design, aim and methodology of the cohort was published in previous papers [19,20].

The study subjects included 252 pregnant women in single pregnancy, not assisted with reproductive technology, recruited from the central part of Poland. All the women with the chronic diseases (including diabetes, hypertension, nephropathy, epilepsy or cancer) and pregnancy complications were excluded from the study. The study subjects were interviewed once in each trimester of pregnancy in order to collect and update sociodemographic data, medical, and reproductive history as well as information about environmental, occupational and lifestyle factors. In addition, during each visit and at delivery, biological samples (saliva and blood) were collected.

One and two years after the children’s birth, the mothers were invited to the medical center where their children’s health status was examined by pediatricians and allergists. For the appropriate recognition of the children’s health status, a questionnaire was administered to the mothers and it was supplemented with information from the medical chart of each child [20,21]. The questionnaire was developed by an allergist based on the recommendations from the International Study of Asthma and Allergies in Childhood. The children were included either to individual allergy type groups or to a healthy group depending on the answer to the following questions: “Has your child ever diagnosed by a doctor with atopic dermatitis?”, “Has your child ever been diagnosed by a doctor with food allergy?”, “Has your child ever wheezed?”

The study was approved by the Ethical Committee of the Nofer Institute of Occupational Medicine, Lodz, Poland (Decision No. 7/2007 and 3/2008). All the women who had agreed to participate in the study were informed about the aims and procedures of the study and were asked to sign an informed consent form. 

### 2.2. Sample Collection and Processing

At each time-point (the 1st trimester of pregnancy, at delivery), 10-mL blood samples were collected into S-MONOVETTE test tubes with lithium heparin as anticoagulant. The blood samples were centrifuged, plasma was collected and stored at −70 °C until the analysis. 

Carotenoids (β-carotene, vitamin A) and E levels were determined by the use of an HPLC system integrated with a 190–800 nm UV-VIS detector [22]. NIST-968e (USA, Fat soluble vitamins, carotenoids in human serum) were used as a standard.

Cotinine levels in saliva of the pregnant women (a biomarker of tobacco smoke exposure) were determined using the high performance liquid chromatography coupled with tandem mass spectrometry (HPLC-ESI + MS/MS) as described and optimized by Stragierowicz et al. [23]. Cotinine concentration of 10 ng/mL was a cut-off value for active smoking and 1.5 ng/mL for passive smoking [24].

### 2.3. Covariates 

The following covariates (based on existing literature and our previous assessments within REPRO_PL cohort) were considered: living area (urban, rural), housing condition (molds at home, home dampness, cleaning frequency, pets at home, cockroaches), parental atopy, child gender, birth outcomes, breastfeeding, and socio-demographic variables (parental age and education, marital status, socio-economic status). The detailed description of the variables was published previously [25].

### 2.4. Statistical Analysis 

The statistical analysis was performed using the Statistica data analysis software system, version 12 (StatSoft, Tulsa, OK, USA). Before the statistical analysis, normality of the data was tested using the Shapiro–Wilk statistic. Variables with normal distribution are presented as mean and standard deviation (SD). Statistical analyses were performed using the one-way analysis of variance (ANOVA) test, and the least significant difference (LSD) post hoc test to compare differences between all the groups.

A simple linear correlation between some analyses was evaluated by the Pearson correlation. *p* value less than 0.05 was considered to be statistically significant.

Finally, the multivariable logistic regression stratified by smoking status during pregnancy was applied. The following variables (significant at 0.1 level) were included into the multivariable model: molds at home, cleaning frequency, pets at home, socio-economic status, maternal education, child gender, atopy in the family, breast feeding and living area. 

## 3. Results

### 3.1. Maternal and Child Characteristics

Table 1 shows characteristic of the pregnant women and their neonates. Mean age of the women at birth was 29 ± 4 years, more than 80% of the women lived in urban areas. Most of the women were highly educated (69%) and with a medium to high socio-economic status (92%). Based on the cotinine level in saliva, about 13% of the women were classified as active and 31% as passive smokers. Maternal smoking resulted in a lower birth weight (3314 ± 480 g vs. 3444 ± 487 g, *p* < 0.05), lower child length (54.3 ± 2.7 cm vs. 55.3 ± 2.8 cm, *p* < 0.009) and lower head circumference (34.1 ± 1.6 cm vs. 34.6 ± 1.6 cm, *p* < 0.025). Visible molds at home were declared by about 9% of the mothers. About 30% of the families kept some animals at home. 

Table 2 shows the children’s health status. About 19% of the children were diagnosed with food allergy and 13% of them with atopic dermatitis. Wheezing was identified in 11% of the children at 1 and 16% of the children at 2 years of age. The allergy symptoms at the age of 1 and 2 was identified for 11%, 7% and 5% of children for food allergy, atopic dermatitis and wheezing, respectively.

### 3.2. Vitamin Concentration 

Cord plasma vitamin concentrations were significantly lower than their levels in maternal plasma in the 1st trimester and at delivery (*p* < 0.001) (Table 3). Significantly higher concentrations of vitamin E have been shown to occur during the 1st trimester of pregnancy in plasma of the women who have actively/passively smoked cigarettes compared to the non-smokers (*p* < 0.02). 

Significant linear correlations were found between concentrations of vitamins in plasma collected at different studied periods (Table 4).

### 3.3. Vitamin Levels and Child Allergy

The mean levels of markers of oxidative stress in the 1st trimester of pregnancy, at delivery and umbilical cord blood in mothers whose children had been diagnosed in the 1st or the 2nd year of life with atopic dermatitis, food allergy or wheezing and healthy children is presented in the Supplementary Materials (Table S1). Table S2 presents the results stratified by maternal smoking status during pregnancy (active/passive smokers and non-smokers).

A significantly higher concentration of β-carotene in the 1st trimester of pregnancy was detected in plasma of the women who were active/passive smokers as compared to the non-smokers with healthy children (0.30 ± 0.20 μg/mL vs. 0.23 ± 0.16 μg/mL, *p* < 0.02) (Table S2). In the perinatal period (maternal blood at delivery), β-carotene concentration was significantly lower in the passive/active smokers whose children developed food allergy in the 1st and 2nd year of their life, compared to the mothers of healthy children (0.25 ± 0.20 μg/mL vs. 0.40 ± 0.25 μg/mL *p* < 0.03 and 0.19 ± 0.14 μg/mL vs. 0.36 ± 0.27 μg/mL *p* < 0.05). The mothers who smoked at any time during pregnancy and whose children developed food allergy in the 2nd year of their life had significantly lower levels of β-carotene than the non-smoking mothers of unhealthy children both in the 1st trimester of pregnancy (0.15 ± 0.08 μg/mL vs. 0.30 ± 0.19 μg/mL, *p* < 0.01) and at delivery (0.19 ± 0.14 μg/mL vs. 0.43 ± 0.22 μg/mL, *p* < 0.02).

Significantly lower levels of vitamin A were found in the 1st trimester of pregnancy in the non-smoking women whose 2-year-old children developed atopic dermatitis (0.82 ± 0.21 μg/mL vs. 0.99 ± 0.30 μg/mL, *p* < 0.004) as compared to the healthy children. In the perinatal period, regardless of tobacco smoke exposure of the pregnant women, significantly lower concentrations of vitamin A have been reported in the women whose children at 2 years of age were diagnosed with atopic dermatitis (maternal blood at delivery: for the non-smokers: 0.76 ± 0.23 μg/mL vs. 0.94 ± 0.36 μg/mL, *p* < 0.02, for the smokers: 0.64 ± 0.29 μg/mL vs. 0.91 ± 0.30 μg/mL, *p* < 0.04; cord blood: for the non-smokers: 0.47 ± 0.20 μg/mL vs. 0.59 ± 0.20 μg/mL, *p* < 0.008; for the smokers: 0.39 ± 0.20 μg/mL vs. 0.60 ± 0.39 μg/mL, *p* < 0.008). Significantly lower plasma levels of vitamin A were detected in the 1st trimester of pregnancy in the women who smoked and whose children developed wheezing during the first year of life, compared to those who did not smoke and whose children were diagnosed with wheezing (0.81 ± 0.25 μg/mL vs. 1.08 ± 0.26 μg/mL, *p* < 0.03).

In the perinatal period (maternal blood at delivery), significantly higher levels of vitamin E were detected in plasma of the pregnant non-smoking women whose children developed atopic dermatitis as compared to the mothers of healthy children (15.65 ± 4.980 μg/mL vs. 11.68 ± 6.66 μg/mL, *p* < 0.006). The concentrations of vitamin E in the active/passive smoking pregnant women, whose one-year-old children developed food allergy were significantly lower than in the mothers of children without allergies (9.59 ± 6.36 μg/mL vs. 14.67 ± 6.05 μg/mL, *p* < 0.01). Significantly higher levels of vitamin E were found in the perinatal period in the non-smoking mothers of children diagnosed with food allergy at 2 years of age than in those mothers whose children were not diagnosed with allergy at 2 years of age (15.78 ± 5.01 μg/mL vs. 11.83 ± 6.83 μg/mL, *p* < 0.03).During the perinatal period (maternal blood at delivery), in plasma of the active/passive smoking pregnant women whose children developed wheezing at the age of 2, levels of vitamin E were significantly higher compared to the mothers of healthy children (16.45 ± 4.80 μg/mL vs. 11.29 ± 6.48 μg/mL, *p* < 0.02).

### 3.4. Association between Markers of Oxidative Stress during Pregnancy and the Risk of Child Allergy—The Multivariable Analysis Stratified by Maternal Smoking Status

The results of multivariable logistic regression analysis for the association between markers of oxidative stress and the risk of allergy at the age of 1 and 2 stratified by maternal active/passive smoking status during pregnancy are presented in Table 5. After inclusion of a variety of confounding factors such as: molds at home, cleaning frequency, pets at home, maternal socio-economic status and education, child gender, parental atopy, breast feeding and living area none of the results were statistically significant (*p* > 0.05). There was no interaction between smoking (active/passive) during pregnancy and vitamins levels on the risk of allergy (*p* > 0.4).

## 4. Discussion

The study investigated the levels of β-carotene and vitamins A and E in plasma of the pregnant women in the 1st trimester of pregnancy and at delivery in relation to allergy development in their children up to 2 years of age. After inclusion of a variety of confounding factors such as: molds at home, cleaning frequency, pets at home, maternal socio-economic status and education, child gender, parental atopy, breast feeding and living area none of the results were statistically significant. The interaction between smoking during pregnancy and vitamins levels on the risk of allergy was not statistically significant.

Oxidative stress accompanies many pathological conditions, including those involving many inflammatory conditions, such as allergies. Oxidative stress is generated during normal physiological processes, but can be also a result of exposure to chemical, biological or physical agents. It is believed that a number of environmental factors affecting pregnant women may contribute to the development of allergy in their children. The potential role of antioxidants, especially those with low molecular weight and provided with a diet, as factors that reduce the risk of developing allergies has been extensively discussed. It has been a subject of numerous studies and raised interest among researchers on the relationship between the intake of low-molecular-weight antioxidants such as vitamins A, C, E and allergy development. 

The results of epidemiological studies are not conclusive: most research confirms beneficial associations between maternal dietary antioxidants intake during pregnancy or maternal plasma levels and allergy outcomes in their children (wheezing, asthma, atopic sensitization), but reports on potentially adverse associations are also accessible [26]. Currently, two contradictory hypotheses concerning the relationship between antioxidants and development of allergies are proposed: too low consumption of vitamins resulting from a change of dietary habits, the way of storing food or preparing meals and the second one: using vitamin supplements or consuming antioxidant rich foods [2,26]. The crucial point in that type of analyses is the inclusion of confounding factors which can be responsible of the observed associations. As it was demonstrated in current study after inclusion of covariates the results were not statistically significant.

Vitamin E is a major lipid-soluble antioxidant directly involved in inhibiting propagation of lipid peroxidation. Changes in plasma concentration of vitamin E during pregnancy are not clearly defined; Chechlowska et al. have shown a significant increase in the concentration of vitamin E during the perinatal period in relation to the first trimester of pregnancy [27]. Smedts et al. have reported that vitamin E significantly increased throughout pregnancy [28]. In our study we found significantly higher concentrations of vitamin E in plasma of the tobacco smoke exposed women in the first trimester of pregnancy compared to the non-exposed women. However, in the perinatal period, there was no difference in the plasma concentration of vitamin E between the tobacco smoke exposed and non-exposed women. Chechlowsk et al. have reported significantly higher levels of vitamin E in the third trimester of pregnancy in smoker women [27]. Other authors have reported lower concentrations of vitamin E in smokers only in the third trimester [29] or no differences [11].

Carotenoids (β-carotene and vitamin A) are also involved in the antioxidant defense, but their contribution to removing ROS is less effective. There were no differences in concentrations of carotenoids between the 1st trimester of pregnancy and the perinatal period and, depending on tobacco smoke exposure, Chechlowska et al. have reported significantly lower vitamin A levels in plasma of the smoker women in all trimesters of pregnancy [27].

During pregnancy, all necessary nutrients are transported through the placenta to a developing fetus [30]. Probably they are immediately transferred to actively metabolizing tissues of the fetus, hence concentrations of the substances essential for proper functioning of the fetus are much lower in the cord than in the maternal blood. Cord blood levels of β-carotene, vitamins A and E are significantly lower (20%, 59% and 40%) than the respective maternal blood plasma levels. Similar findings have been reported by other authors [30,31].

In our study, concentration of the cord plasma vitamin A is equal to 59% of vitamin A concentration in the plasma of the women at birth. This is in agreement with the data reported by other authors which indicates that the cord vitamin A level varies from 50–60% of the maternal values [32].

Exposure to tobacco smoke during pregnancy has been an important public health problem for many decades. Analysis of the data published throughout nearly 20 years indicates that, despite some geographical and cultural differences, approx. up to 30% of female smokers continue to smoke cigarettes during pregnancy and after childbirth [33]. The analysis on Polish Mother and Child Cohort conducted in a group of 1771 pregnant women indicated that about 15% of pregnant women smoked cigarettes, while about 35% was exposed to tobacco smoke at home [24]. Maternal smoking during pregnancy is associated with an elevated risk of pregnancy (pregnancy induced hypertension, preeclampsia, gestational diabetes etc.) and with adverse fetal outcomes (intrauterine fetal growth retardation and with disturbances in postnatal growth and development) [34,35]. Analysis of pre- and postnatal tobacco smoke exposure and development of respiratory illness during childhood points to the origins of respiratory disease during fetal life [33]. Tobacco smoke contains more than 5000 chemicals, many of which have proven adverse effects on human health [36]. Their association with an increased risk of developing asthma and other respiratory diseases is also proven [37]. Research carried out nearly 20 years ago showed no association between parental smoking during pregnancy and an increased positive response to allergen skin prick tests (OR = 0.87, 95% CI 0.62–1.24) [38]. However, a multicenter study carried out in Germany has shown that children prenatally exposed to environmental tobacco smoke had a higher probability of sensitization to food allergens (OR = 2.3, 95% CI 1.1–4.6) [39].

Lee has found that adult onset of atopic dermatitis was associated with gestational and prenatal exposure to tobacco smoke [40]. Tobacco smoke may affect immunological processes—newborns of smoking mothers have a diminished innate immune response. 

Based on the analysis of the studied population and changes in the concentration of antioxidant vitamins during pregnancy and their relationship with the occurrence of allergies in young children, it was concluded that lower concentrations of vitamin A were observed in the women whose children developed respiratory and skin allergies. It is characteristic that statistically significant differences were found in maternal blood during the perinatal period and only respiratory allergies were associated with exposure to tobacco smoke.

Vitamin A and pro-vitamin A carotenoids (e.g., α-carotene, β-carotene, lutein, zeaxantin) are necessary for normal growth and homeostasis of organisms. Vitamin A plays a crucial role in immune functions. In vitro studies suggest that deficiency of antioxidants interferes with differentiation and promotion of T-helper cells, i.e., cells responsible for safeguarding the immune function [41]. The research of Maslova et al. shows that high maternal intake of vitamin A reduces the risk of allergy in children [42]. However, this inverse relationship is associated with natural sources of vitamin A: vegetables and fruit, milk, and not vitamin A itself. So the risk of allergy is reduced by the intake of carotenoids, and not of chemically pure vitamin A. Therefore, the immune response is responsible for a path of metabolism, in which carotenoids are incorporated. The Polish study group of pregnant women had higher levels of vitamin A and β-carotene than pregnant cohort study in Scotland [30]. Women in Poland often use multivitamin dietary supplements. The concentrations determined in our population are the sum of vitamins supplied with a diet (vitamin A, pro-vitamin A, other carotenoids) and supplements (probably vitamin A). Supplements may contain vitamin A, with a small amount of pro-vitamin A carotenoids, hence, they cannot fulfill a protective role against allergy development. It is also important that the concentration of carotenoids in plasma that is needed to decrease inflammation in target organs during pregnancy is variable [43]. Therefore, they constitute a labile pool used for the current metabolic needs. Also results of the analysis of the relationship between vitamin E in the life of prenatal allergies in the children of 2 years of age are ambiguous. High maternal intake of vitamin E during pregnancy reduces the risk of asthma and wheezing in children at the age 1–2 years. No association has been found with eczema [44,45].

The impact of vitamins intake during pregnancy and the incidence of allergies in children depends certainly on basal vitamins intake. Research shows that the average intake of vitamins E is 7.8 mg/d in Japan, 30.7 mg/d in the US, 8.2 mg/d in the UK and 12.3 mg/d in Sweden [41]. In Poland the recommended daily intake of vitamin E is 10 mg/d. In the studies from 2011–2012 it was demonstrated that about 15% of the general population use dietary supplements. Concentration of vitamin E in supplement users may amount even up to 300% of the daily recommended dose [46]. Frequently neither this supplementation is justified by medical issues nor does it result from medical indications. The same concerns pregnant women. Analysis of vitamin intake in pregnant women living in central Poland assessed by a 24-h recall interview repeated 3 times for each study woman, has indicated that the intake of vitamin E exceeded the recommended daily allowance level by 70%, vitamin A—by 30%. This is probably due to the widespread use of vitamin supplements by pregnant women. Vitamin and mineral supplementation is practiced by 80–90% of pregnant women, both in the period immediately before pregnancy and during pregnancy itself [47]. Probably such a different vitamin E intake can be a critical factor contributing to the risk of allergies development in children. The differences concerning timing of assessment of dietary intake have small effect on the risk of allergies in small children [48]. As a result there are discrepancies in the results of the studies published by various research centers looking for a relationship between vitamins A and E intake and the allergy risk in small children. Therefore, it seems that assessment of concentrations of antioxidant vitamins in plasma is a more appropriate marker serving for the purpose of the study of the role of antioxidants and allergy development [49]. Finally as it was stated above the inclusion of variety confounding factors is the crucial point in the analysis of the impact of vitamins on the risk of child allergy. 

The strength of the study is the prospective study design and assessment of vitamins concentrations at different time points (1st trimester, maternal blood at delivery, cord blood). In addition, active/passive smoking status of pregnant women was assessed based on a biomarker of exposure. The main limitation of the present study is the fact that we looked only at prenatal exposure and were not able to evaluate vitamins levels in children. Taking into account children’s age (up to 2 years of age) neither skin prick testing nor pulmonary function testing were performed. However, it needs to be pointed out that the children from REPRO_PL cohort are currently the subjects of assessment at the age of 7 years (with these tests included in children’s health status examination). This will allow a more objective outcome evaluation in future research.

## 5. Conclusions

Multivariate analysis with inclusion of a variety of confounding factors have not indicated any statistically significant associations between β-carotene and vitamins A and E in plasma of the pregnant women in the 1st trimester of pregnancy and at delivery in and the risk of food allergy, atopic dermatitis and wheezing in their children up to 2 years of age. There was no interaction between smoking during pregnancy and vitamins levels on the risk of allergy.

## Figures and Tables

**Table 1:** Characteristics of the pregnant women and anthropometric measures of their neonates.

Characteristic	*n* = 252
Mothers’ age, years. (*n* = 248)	29.1 ± 4.2
Fathers’ age, years. (*n* = 227)	31.2 ± 5.3
Living area (*n* = 251)	
Rural	47 (18.7%)
Urban	204 (81.32%)
Mothers’ education	
Primary	10 (4.0%)
Secondary	69 (27.4%)
University	173 (68.6%)
Socio-economic status	
Low	21 (8.3%)
Medium	198 (78.6%)
High	33 (13.1%)
Mothers’ BMI (kg/m^2^)	
Prepregnancy (*n* = 242)	23.2 ± 3.7
1st trimester (*n* = 233)	23.2 ± 3.8
2nd trimester (*n* = 227)	224.7 ± 3.9
3rd trimester (*n* = 209)	226.8 ± 4.0
Parental atopy	
Yes	45 (17.9%)
No	207 (82.1%)
Maternal smoking	
Ever smokers	90 (35.7%)
Smokers during pregnancy (saliva cotinine level ≥10 ng/mL)	32 (12.7%)
Never smokers	156(61.9%)
Passive smokers during pregnancy(saliva cotinine level ≥1.5 ng/mL)	78 (30.9%)
No data	6 (2.4%)
Molds at home	
Yes	23 (9.1%)
No	229 (90.9%)
Pets at home (*n* = 237)	
Yes	70 (29.5%)
No	167 (70.5%)
Child gender	
Male	125 (49.6%)
Female	127 (50.4%)
Breast feeding	
No	26 (10.3%)
Yes	226 (89.7%)
≤6 months	111 (49.4%)
>6–≤12 months	78 (34.7%)
No data	35 (15.7%)
Gestational age (weeks) (*n* = 248)	39.2 ± 1.4
Birthweight (g) (*n* = 237)	3394 ± 486
Body length (cm) (*n* = 237)	55 ± 3

**Table 2:** The children’s health status.

	Total Number of the Examined Children 1-Year Old/2-Year Old	Number of the Children Diagnosed with Allergy within 1 Year of Their Lifetime	Number of the Children Diagnosed with Allergy within 2 Years of Their Lifetime	Number of the Children Diagnosed with Allergy within the 1st and 2nd Year of Their Lifetime
Food allergy	221/160	43 (19.5%)	30 (18.7%)	17 (10.6%)
Atopic dermatitis	252/252	34 (13.5%)	32 (12.7%)	18 (7.1)
Wheezing	217/167	23 (10.6%)	27 (16.2%)	8 (4.8)

**Table 3:** β-Carotene, vitamin A and E concentrations in plasma in the pregnant women in the 1st trimester, at delivery and in umbilical cord plasma.

Plasma Vitamins Level	Total, *n* = 252	Passive and Active Smokers, *n* = 90	Non-Smokers, *n* = 156
β-Carotene, μg/mL			
1st trimester	0.252 ± 0.185	0.277 ± 0.195	0.238 ± 0.174
at delivery	0.330 ± 0.222 *	0.337 ± 0.240	0.329 ± 0.207
Cord blood	0.051 ± 0.045 *^,&^	0.053 ± 0.053 *^,&^	0.050 ± 0.039 *^,&^
Vitamin A, μg/mL			
1st trimester	0.954 ± 0.275	0.949 ± 0.254	0.963 ± 0.290
at delivery	0.896 ± 0.334	0.888 ± 0.306	0.908 ± 0.347
Cord blood	0.569 ± 0.204 *^,&^	0.578 ± 0.200 *^,&^	0.571 ± 0.202 *
Vitamin E, μg/mL			
1st trimester	8.67 ± 3.65	9.38 ± 3.43	8.21 ± 3.71 ^#^
at delivery	12.50 ± 6.63	12.94 ± 6.33	12.37 ± 6.70
Cord blood	3.44 ± 2.17 *^,&^	3.73 ± 2.57 *^,&^	3.33 ± 1.86 *^,&^

* statistically significant as compared with the 1st trimester, *p* < 0.001; ^&^ statistically significant as compared with maternal blood at delivery time, *p* < 0.001; ^#^ statistically significant as compared with the passive and active smokers, *p* < 0.02.

**Table 4:** Correlation between plasma vitamins A, E and β-carotene during the 1st trimester of pregnancy, at delivery and in cord blood plasma.

	Correlation Coefficient		
	1st Trimester/at Delivery	at Delivery/Cord Blood	1st Trimester/Cord Blood
Vitamin A	0.75; *p* < 0.0001	0.730; *p* < 0.0001	0.660; *p* < 0.0001
β-Carotene	0.56; *p* < 0.0001	0.45; *p* < 0.0001	0.42; *p* < 0.0001
Vitamin E	0.41; *p* < 0.0001	0.45; *p* < 0.0001	0.32; *p* < 0.0001

**Table 5:** Association between markers of oxidative stress in the 1st trimester of pregnancy, at delivery and umbilical cord blood stratified by maternal smoking status (active/passive smokers, non-smokers) and the risk of atopic dermatitis, food allergy or wheezing at the age of 1 and 2—the multivariable model.

	One Year Old Children			Two Year Old Children		
	Food Allergy	Atopic Dermatitis	Wheezing	Food Allergy	Atopic Dermatitis	Wheezing
	OR (95% CI)					
**Active or Passive Smokers during Pregnancy**						
**β-Carotene, µg/mL**						
1st trimester	1.14 (0.06–22.57)	0.45 (0.01–13.14)	0.06 (0.01–32.72)	0.27 (0.01–48.36)	0.55 (0.02–13.33)	1.56 (0.01–487.3)
Delivery	0.03 (0.01–1.75)	1.55 (0.06–40.94)	0.20 (0.01–1.50)	0.09 (0.01–15.78)	0.39 (0.02–9.44)	16.94 (0.06–465.0)
Cord blood	1.54 (0.28–8.29)	3.67 (0.85–9.63)	0.05 (0.01–40.75)	6.43 (0.03–164.3)	9.39 (0.21–48.01)	0.14 (0.01–179.0)
**Vitamin A, µg/mL**						
1st trimester	1.02 (0.08–12.58)	0.53 (0.06–15.08)	0.47 (0.01–17.07)	0.4 (0.01–25.55)	0.27 (0.02–3.67)	5.18 (0.06–485.0)
Delivery	0.57 (0.05–1.99)	0.86 (0.06–7.12)	2.68 (0.12–61.09)	1.98 (0.13–29.32)	0.17 (0.02–1.74)	1.56 (0.07–36.06)
Cord blood	1.42 (0.04–45.12)	0.29 (0.01–12.72)	4.63 (0.02–12.18)	5.85 (0.03–105.79)	0.07 (0.01–2.52)	16.92 (0.13–228.6)
**Vitamin E, µg/mL**						
1st trimester	1.03 (0.86–1.24)	0.95 (0.78–1.16)	0.99 (0.77–1.26)	0.98 (0.80–1.21)	0.91 (0.76–1.08)	1.11 (0.87–1.41)
Delivery	0.85 (0.74–1.12)	1.05 (0.94–1.19)	0.91 (0.75–1.09)	1.07 (0.93–1.24)	0.99 (0.89–1.10)	1.02 (0.86–1.22)
Cord blood	1.05 (0.75–1.45)	1.55 (0.82–2.36)	0.35 (0.12–1.09)	4.95 (0.98–17.75)	1.35 (0.96–1.89)	1.18 (0.75–1.86)
**Non-Smokers during Pregnancy**						
**β-Carotene, µg/mL**						
1st trimester	4.70 (0.30–74.00)	6.29 (0.37–96.56)	0.54 (0.01–22.42)	0.29 (0.01–10.98)	4,97 (0.22–113.8)	0.02 (0.01–2.18)
Delivery	1.18 (0.12–11.41)	1.57 (0.13–19.76)	0.75 (0.03–20.79)	5.17 (0.26–103.9)	2.87 (0.29–28.55)	1.41 (0.07–29.38)
Cord blood	0.18 (0.02–3.41)	0.13 (0.04–9.16)	0.71 (0.01–9.87)	0.01 (0.01–485.0)	0.03 (0.01–55.22)	0.18 (0.01–32.36)
**Vitamin A, µg/mL**						
1st trimester	1.44 (0.21–9.63)	0.38 (0.04–3.27)	1.52 (0.17–13.92)	0.12 (0.01–1.63)	0.03 (0.01–1.36)	0.47 (0.05–4.19)
Delivery	0.59 (0.12–3.05)	0.29 (0.05–1.73)	0.18 (0.02–1.97)	0.29 (0.03–3.10)	0.08 (0.01–1.66)	0.38 (0.05–3.22)
Cord blood	0.09 (0.01–1.43)	0.21 (0.01–4.35)	0.15 (0.01–4.62)	0.03 (0.01–1.68)	0.06 (0.01–1.23)	0.10 (0.01–4.23)
**Vitamin E, µg/mL**						
1st trimester	1.11 (0.93–1.28)	1.18 (0.91–1.38)	1.11 (0.94–1.32)	1.14 (0.97–1.35)	1.01 (0.86–1.18)	1.00 (0.85–1.16)
Delivery	1.01 (0.93–1.09)	1.08 (0.98–1.18)	1.04 (0.93.1.18)	1.12 (1.00–1.27)	1.03 (0.94–1.14)	1.07 (0.96–1.20)
Cord blood	0.88 (0.66–1.17)	1.03 (0.79–1.33)	1.52 (0.92–2.12)	1.04 (0.74–1.47)	1.04 (0.76–1.42)	1.06 (0.74–1.52)

Adjusted for: molds at home, cleaning frequency, pets at home, maternal socio-economic status and education, child gender, parental atopy, breast feeding and living area. *p* for interaction >0.4.

## Supplementary Materials

The following are available online at http://www.mdpi.com/1660-4601/15/6/1245/s1, Table S1: Markers of oxidative stress in the 1st trimester of pregnancy, at delivery and umbilical cord blood in mothers whose children had been diagnosed in the 1stor the 2nd year of lifewith atopic dermatitis, food allergy or wheezing and healthy children (*t* test), Table S2: Markers of oxidative stress in the 1st trimester of pregnancy, at delivery and umbilical cord blood in smoking and non-smoking mothers whose children had been diagnosed in the 1st or the 2nd year of lifetime with atopic dermatitis, food allergy or wheezing and healthy children (*t* test).
