# Supplementary material for: Vitamins A and E during Pregnancy and Allergy Symptoms in an Early Childhood—Lack of Association with Tobacco Smoke Exposure
Source: Int J Environ Res Public Health. 2018 Jun 12;15(6):1245. doi: 10.3390/ijerph15061245 (PMC6025152; doi:10.3390/ijerph15061245)
Supplement: Supplementary file 1 [file ijerph-15-01245-s001.pdf]

Table S1. Markers of oxidative stress in the 1st trimester of pregnancy, at delivery and umbilical cord blood in mothers whose children had been diagnosed in the 1st or the 2nd year of life with atopic dermatitis, food allergy or wheezing and healthy children (t test).

	**Healthy Children 1st Year**	**Healthy Children 2nd Year**	**Atopic Dermatitis 1st Year**	**Atopic Dermatitis 2nd Year**	**Atopic Dermatitis 1st and 2nd Year**	**Healthy Children 1st Year**	**Healthy Children 2nd Year**	**Food Allergy 1st Year**	**Food Allergy 2nd Year**	**Food Allergy 1st and 2nd Year**	**Healthy Children 1st Year**	**Healthy Children 2nd Year**	**Wheezing 1st Year**	**Wheezing 2nd Year**	**Wheezing 1st and 2nd Year**
**B-carotene, μg/ml<br>Mean ± SD**															
**1st trimester**	0.25 ± 0.18	0.25 ± 0.18	0.30 ± 0.20	0.27 ± 0.19	0.33 ± 0.23	0.26 ± 0.18	0.26 ± 0.18	0.29 ± 0.20	0.23 ± 0.19	0.29 ± 0.22	0.23 ± 0.18	0.26 ± 0.19	0.23 ± 0.15	0.21 ± 0.12	0.24 ± 0.10
**Delivery**	0.32 ± 0.22	0.33 ± 0.22	0.37 ± 0.23	0.35 ± 0.22	0.40±0.26	0.36 ± 0.22	0.33 ± 0.22	0.33 ± 0.22	0.33 ± 0.22	0.37 ± 0.28	0.36 ± 0.23	0.33 ± 0.22	0.33 ± 0.19	0.35 ± 0.20	0.46 ± 0.15
**Cord blood**	0.05 ± 0.05	0.05 ± 0.05	0.05 ± 0.04	0.05 ± 0.04	0.06±0.05	0.06 ± 0.05	0.05 ± 0.04	0.05 ± 0.04	0.05 ± 0.04	0.06 ± 0.05	0.05 ± 0.05	0.05 ± 0.04	0.06 ± 0.06	0.04 ± 0.02	0.06 ± 0.02
**Vitamin A, ug/ml<br>Mean ± SD**															
**1st trimester**	0.96 ± 0.28	0.98 ± 0.28	0.92 ± 0.27	0.81 ± 0.20<br>**$p < 0.001$**	0.78 ± 0.23	0.94 ± 0.28	0.98 ± 0.27	0.96 ± 0.25	0.86 ± 0.22<br>**$p < 0.04$**	0.84 ± 0.25	0.94 ± 0.27	0.95 ± 0.27	0.96 ± 0.28	0.97 ± 0.30	0.99 ± 0.30
**Delivery**	0.91 ± 0.34	0.92 ± 0.34	0.83 ± 0.31	0.73 ± 0.25<br>**$p < 0.004$**	0.70 ± 0.27	0.86 ± 0.32	0.92 ± 0.35	0.83 ± 0.32	0.84 ± 0.28	0.73 ± 0.26	0.87 ± 0.33	0.90 ± 0.34	0.83 ± 0.25	0.885 ± 0.34	0.74 ± 0.17
**Cord blood**	0.58 ± 0.20	0.59 ± 0.20	0.52 ± 0.22	0.45 ± 0.20<br>**$p < 0.001$**	0.42 ± 0.19	0.57 ± 0.20	0.57 ± 0.20	0.53 ± 0.22	0.52 ± 0.23	0.46 ± 0.24	0.57 ± 0.21	0.56 ± 0.21	0.54 ± 0.15	0.569 ± 0.19	0.49 ± 0.09
**Vitamin E, ug/ml<br>Mean ± SD**															
**1st trimester**	8.50 ± 3.63	8.73 ± 3.63	9.73 ± 3.56	8.23 ± 3.70	8.97 ± 4.00	8.67 ± 3.56	8.55 ± 3.53	9.42 ± 3.48	9.34 ± 4.63	9.87 ± 4.18	8.79 ± 3.64	8.50 ± 3.75	9.57 ± 3.59	9.09 ± 3.97	8.83 ± 3.60
**Delivery**	12.17 ± 6.57	12.51 ± 6.58	16.00 ± 5.85<br>**$p < 0.022$**	12.92 ± 6.37	14.12 ± 6.42	13.58 ± 6.50	12.28 ± 6.65	11.80 ± 6.55	13.99 ± 6.04	13.56 ± 6.55	13.48 ± 6.44	12.01 ± 6.89	13.67 ± 6.47	14.43 ± 4.71	14.61 ± 3.15
**Cord blood**	3.40 ± 2.13	3.43 ± 2.12	3.96 ± 2.14	3.76 ± 2.28	4.35 ± 2.55	3.80 ± 2.20	3.24 ± 1.77	3.44 ± 1.99	3.83 ± 2.35	3.91 ± 2.50	3.69 ± 2.03	3.31 ± 1.99	4.65 ± 3.24	3.48 ± 1.32	4.42 ± 1.32

p—statistically significance as compared with healthy children.

Table S2. Markers of oxidative stress in the 1st trimester of pregnancy, at delivery and umbilical cord blood in smoking and non-smoking mothers whose children had been diagnosed in the 1st or the 2nd year of lifetime with atopic dermatitis, food allergy or wheezing and healthy children (t test).

	Healthy Children 1st Year	Healthy Children 2nd Year	Atopic Dermatitis 1st Year	Atopic Dermatitis 2nd Year	Healthy children 1st Year	Healthy Children 2nd Year	Food Allergy 1st Year	Food Allergy 2nd Year	Healthy Children 1st Year	Healthy Children 2nd Year	Wheezing 1st Year	Wheezing 2nd Year
**B-carotene, μg/ml Mean ± SD**												
**Non-smokers**												
**1st trimester**	0.25 ± 0.18	0.26 ± 0.19	0.39 ± 0.21	0.32 ± 0.17	0.23 ± 0.16	0.23 ± 0.16	0.36 ± 0.22	0.30 ± 0.19 $p < 0.02$	0.27 ± 0.19	0.29 ± 0.20	0.27 ± 0.19	0.19 ± 0.07
**Delivery**	0.34 ± 0.25	0.34 ± 0.25	0.42 ± 0.26	0.42 ± 0.20	0.36 ± 0.25	0.34 ± 0.25	0.43 ± 0.25	0.43 ± 0.22	0.37 ± 0.26	0.36 ± 0.26	0.37 ± 0.26	0.35 ± 0.19
**Cord blood**	0.06 ± 0.05	0.06 ± 0.05	0.05 ± 0.03	0.05 ± 0.02	0.06 ± 0.06	0.05 ± 0.04	0.05 ± 0.03	0.06 ± 0.03	0.06 ± 0.05	0.06 ± 0.04	0.06 ± 0.05	0.05 ± 0.02
**Active or passive smokers during pregnancy**												
**1st trimester**	0.24 ± 0.18	0.24 ± 0.18	0.25 ± 0.17	0.23 ± 0.17	0.30 ± 0.20 $p < 0.02$ *	0.24 ± 0.16	0.25 ± 0.20	0.15 ± 0.08 $p < 0.014$ *	0.27 ± 0.18	0.24 ± 0.17	0.22 ± 0.11	0.22 ± 0.14
**Delivery**	0.31 ± 0.19	0.32 ± 0.20	0.36 ± 0.21	0.29 ± 0.19	0.40 ± 0.25	0.36 ± 0.27	0.25 ± 0.20 $p < 0.03$	0.19 ± 0.14 $p < 0.05$ $p < 0.02$ *	0.34 ± 0.20	0.30 ± 0.19	0.32 ± 0.18	0.36 ± 0.21
**Cord blood**	0.04 ± 0.04	0.05 ± 0.04	0.06 ± 0.05	0.05 ± 0.05	0.05 ± 0.04	0.05 ± 0.04	0.04 ± 0.04	0.05 ± 0.05	0.05 ± 0.04	0.05 ± 0.05	0.04 ± 0.02	0.04 ± 0.02
**Vitamin A, ug/ml Mean ± SD**												
**Non-smokers**												
**1st trimester**	1.01 ± 0.31	0.99 ± 0.30	0.83 ± 0.27	0.82 ± 0.21 $p<0.004$	0.99 ± 0.31	0.97 ± 0.29	0.96 ± 0.26	0.98 ± 0.23	0.95 ± 0.29	0.96 ± 0.29	1.08 ± 0.26	0.94 ± 0.38
**Delivery**	0.20 ± 0.37	0.94 ± 0.36	0.72 ± 0.35	0.76 ± 0.23 $p<0.02$	0.92 ± 0.35	0.98 ± 0.39	0.91 ± 0.36	0.83 ± 0.28	0.93 ± 0.35	0.95 ± 0.38	0.74 ± 0.28	0.83 ± 0.49
**Cord blood**	0.59 ± 0.20	0.59 ± 0.20	0.47 ± 0.28	0.47 ± 0.20 $p<0.008$	0.60 ± 0.22	0.58 ± 0.20	0.53 ± 0.23	0.49 ± 0.27	0.59 ± 0.23	0.57 ± 0.22	0.52 ± 0.14	0.48 ± 0.17
**Active or passive smokers during pregnancy**												
**1st trimester**	0.93 ± 0.25	0.96 ± 0.26	0.93 ± 0.28	0.79 ± 0.20	0.91 ± 0.25	0.83 ± 0.24	0.95 ± 0.25	0.92 ± 0.45	0.94 ± 0.24	0.94 ± 0.25	0.81 ± 0.25* $p < 0.03$	0.98 ± 0.27

**Delivery**	0.87 ± 0.29	0.91 ± 0.30	0.84 ± 0.30	0.64 ± 0.29 $p < 0.04$	0.82 ± 0.28	0.90 ± 0.30	0.79 ± 0.30	0.85 ± 0.29	0.81 ± 0.29	0.87 ± 0.31	0.88 ± 0.23	0.91 ± 0.26
**Cord blood**	0.58 ± 0.19	0.60 ± 0.39	0.51 ± 0.19	0.39 ± 0.20 $p < 0.008$	0.55 ± 0.19	0.58 ± 0.19	0.53 ± 0.21	0.54 ± 0.21	0.55 ± 0.20	0.56 ± 0.20	0.56 ± 0.17	0.61 ± 0.19
**Vitamin E, ug/ml Mean ± SD**												
**Non-smokers**												
**1st trimester**	7.98 ± 3.67	8.22 ± 3.65	9.67 ± 3.75	8.16 ± 4.12	8.22 ± 3.58	8.09 ± 3.91	9.26 ± 3.77	10.07 ± 5.10	8.33 ± 3.63	8.39 ± 4.12	9.207 ± 3.91	7.93 ± 4.13
**Delivery**	11.68 ± 6.66	11.75 ± 6.63	15.65 ± 4.80 $p < 0.006$	14.64 ± 4.51	13.03 ± 6.70	11.83 ± 6.83	13.89 ± 6.56	15.78 ± 5.01 $p < 0.03$	11.77 ± 6.76	12.25 ± 7.07	16.18 ± 4.94 $p < 0.006$	12.41 ± 3.81
**Cord blood**	3.61 ± 2.57	3.62 ± 2.59	4.03 ± 1.30	3.74 ± 1.28	3.93 ± 2.61	3.27 ± 1.73	3.70 ± 1.65	3.79 ± 1.46	3.65 ± 2.19	3.34 ± 1.71	6.58 ± 4.02	3.45 ± 1.57
**Active or passive smokers during pregnancy**												
**1st trimester**	9.30 ± 3.46	9.45 ± 3.51	9.84 ± 3.34 $p < 0.04$ **	8.46 ± 2.18	9.37 ± 3.45	9.07 ± 3.32	9.72 ± 3.17	8.85 ± 4.38	9.55 ± 3.60	8.59 ± 3.46	10.02 ± 3.31	9.58 ± 3.91
**Delivery**	12.70 ± 6.48	13.26 ± 6.47	15.07 ± 6.50	11.71 ± 6.72	14.67 ± 6.05	12.99 ± 6.33	9.59 ± 6.36 $p < 0.01$	11.39 ± 6.70	12.90 ± 6.27	11.29 ± 6.48	13.22 ± 7.01	16.45 ± 4.80 $p < 0.02$
**Cord blood**	3.24 ± 1.73	3.30 ± 1.69	4.16 ± 2.53	3.77 ± 2.83	3.71 ± 1.80	3.33 ± 1.85	3.30 ± 2.17	3.85 ± 2.79	3.73 ± 1.92	3.27 ± 2.19	3.15 ± 1.35	3.50 ± 1.21

p—statistically significance as compared with healthy children; * *p*— statistically significance as compared with non-smokers; ** *p* –statistically significant as compared with the non-tobacco smoke exposed women who had healthy children.
